# A Circular Economy Approach to Integrate Divergent Ruminant Production Systems: Using Dairy Cow Feed Leftovers to Enhance the Out-of-Season Reproductive Performance in Goats

**DOI:** 10.3390/ani13152431

**Published:** 2023-07-27

**Authors:** Maria G. Machado-Ramos, Cesar A. Meza-Herrera, Angeles De Santiago-Miramontes, Miguel Mellado, Francisco G. Véliz-Deras, Fernando Arellano-Rodríguez, Viridiana Contreras-Villarreal, José R. Arévalo, Dalia I. Carrillo-Moreno, Jessica M. Flores-Salas

**Affiliations:** 1 Programa de Posgrado en Ciencias en Producción Agropecuaria, Universidad Autónoma Agraria Antonio Narro Unidad Laguna, Torreon 27054, Mexico; 2 Unidad Regional Universitaria de Zonas Áridas, Universidad Autónoma Chapingo, Bermejillo 35230, Mexico; 3 Programa de Posgrado en Ciencias en Producción Agropecuaria, Universidad Autónoma Agraria Antonio Narro, Saltillo 25315, Mexico; 4 Department of Botany, Ecology and Plant Physiology, Faculty of Sciences, Universidad de La Laguna, 38200 La Laguna, Spain

**Keywords:** goats, metabolic status, anestrous, male effect, feed surplus, ovulation rate, pregnancy rate

## Abstract

**Simple Summary:**

In the arid lands of Northern Mexico, there are several dairy cattle clusters managed under intensive production systems and fed completely with mixed rations, yet 3–5% feed rejection of the total ration offered has been reported. This surplus feed retains a high nutritional value which could be used to improve the reproductive efficiency of goats managed under marginal–extensive schemes. Therefore, we visualized an interesting research opportunity based on the circular economy approach. We hypothesized that a short-term supplementation with feed leftovers from dairy farms (i.e., intensive system) will increase the reproductive outcomes of rangeland managed goats (i.e., extensive system), trying to promote the circularity/connectedness between two highly discordant production systems. Our study intended to elucidate this working hypothesis.

**Abstract:**

Based on a circular economy approach, we evaluated the possible effect of targeted supplementation with leftover feed from dairy cows (i.e., intensive system) on the reproductive performance of crossbred/rangeland goats (i.e., extensive system) in arid Northern Mexico. During the deep-anestrous season (i.e., March–April, 25° North), multiparous goats (*n* = 38) with a similar body weight (BW) and body condition score (BCS) were randomly assigned to two groups: (1) supplemented group (SG; *n* = 19; BCS: 1.76 ± 0.07; BW: 43.7 ± 1.8 kg), receiving 400 g goat d^−1^ of dairy-cow-feed leftovers prior to grazing; and (2) non-supplemented group (NS; *n* = 19; BCS: 1.76 ± 0.06; BW: 44.3 ± 2.5 kg). Both groups were directed to the rangeland for a period of ≈8 h. While the experimental period lasted 36 d, the experimental breeding considered 11 d (d0–d10). The anovulatory status of goats was ultrasonographically confirmed on days −20, −10, and −1 prior to male–female interaction. Previously, bucks were separated for 3 weeks from the experimental females and received exogenous testosterone every third day (i.e., 50 mg i.m.) prior to mating. With respect to the response variables, namely BW, BCS, blood glucose levels (BGLs), estrus induction (GIE, %), latency to estrus (LTE, h), estrus duration (ED, h), and luteal tissue volume (LTV, mm), no differences (*p* > 0.05) occurred between experimental groups. However, the response variables, namely goats ovulating (GO, %; 78.9 vs. 47.3), total number of corpuses luteum (TNCL, *n*; 27 vs. 13), ovulation rate (OR, *n*; 1.42 vs. 0.73), multiple ovulation (MO, %; 73.3 vs. 55.5), and pregnancy rate on d 36 (PRd36, %, 68.4 vs. 36.8), favored (*p* < 0.05) the SG over the NS goats. Our results demonstrate that connecting the circularity of two divergent ruminant production systems (i.e., cow-intensive and goat-extensive) by using dairy cows’ feed leftovers as a targeted supplementation strategy in anestrous goats under a marginal-rangeland production system enhanced out-of-season reproductive outcomes (i.e., ovulation rate and pregnancy rate), thus benefiting marginal goat producers and their families.

## 1. Introduction

At latitudes >30° N, seasonal sexual activity in goats is determined by the photoperiod, governed by a circannual rhythm involving daylight. Once the photoperiod decreases, the pineal gland induces a more prolonged secretion of melatonin in neurons of the hypothalamic medio basal preoptic area, activating greater GnRH pulsatility [1]. Therefore, in most goats located at latitudes greater than 30° N, while long day photoperiods inhibit sexual activity, short days induce reproductive activity. However, reproductive seasonality may vary according to breed, showing dissimilar reproductive responses at different latitudes; native goats to tropical areas may show low or non-reproductive seasonality [2]. Moreover, sexual behavior and reproductive outcomes can be modulated through hormonal treatments [3], sociosexual interactions [4], hierarchical structures within the herd [5], and nutritional status, which results in different body condition scores (BCS) for the animals [6,7]. In the arid and semi-arid zones of Northern Mexico (25° N), most goats are kept in rangeland, which presents drastic feed fluctuations, either in regard to quantity or quality; therefore, the nutritional supplementation of goats is generally required to maintain an adequate reproductive performance [8].

However, feeding them commercial grains and concentrates is expensive, thus hindering their use by most goat farmers [9] The use of by-products from the food industry has been recognized as a cheap form of supplementation for farm animals; likewise, the non-conventional feeds [6,9] can serve as potential ingredients for feeding goats on rangeland [10]. In the arid lands of Northern Mexico, there are several dairy cattle clusters that are managed under intensive production systems and fed completely with mixed rations [11]. Under this type of nutritional management, 3–5% feed rejection of the total ration offered has been observed [12]. This surplus feed retains a high nutritional value [6], which could be used to improve the reproductive efficiency of goats managed under marginal–extensive schemes. Therefore, we visualized an interesting research opportunity based on the circular economy approach [13]. We aimed to link a small transect observed in the classical highly industrialized large-scale dairy cow clusters settled in the arid lands of Northern Mexico, majorly based on an unsustainable, wasteful linear model of production (i.e., with an inventory (I) of 493,144 Holstein cows, the ecological cost (EC) represents 2340% regarding the Economic Value of Production (EVP)) [14] to the classical extensive goat production system, resembling a more circular, less linear, more closed loop (i.e., with an I = 390,423 crossbred goats, the EC represents 32.6% regarding the EVP) [15]. We hypothesized that a short-term supplementation with feed leftovers from dairy farms (i.e., intensive system) would increase the reproductive outcomes of rangeland-managed goats (i.e., extensive system), trying to promote the circularity between two highly discordant production systems. This study was designed to test this working hypothesis.

## 2. Materials and Methods

### 2.1. General

All experimental procedures, methods, and handling of experimental units used in this study complied with international [16] and national [17] standards for the ethical use, care, and welfare of animals in research, with institutional approval reference number UAAAN-UL-13-10 8242 2641.

### 2.2. Study Area and Environmental Conditions

**Figure 1:**
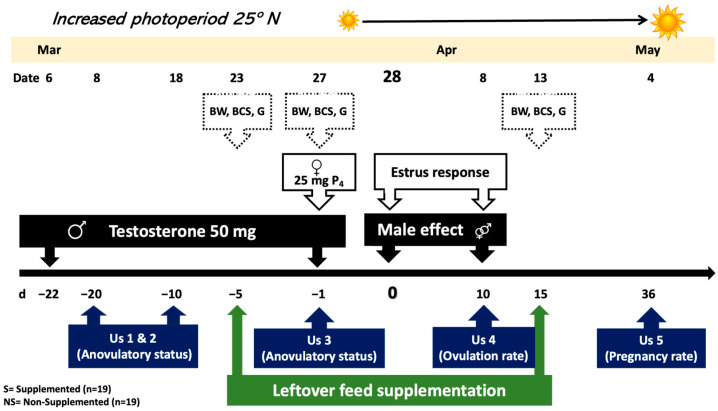
Schematic representation of the experimental protocol in of multiparous crossbred/rangeland goats (*n* = 38) (i.e., extensive system) receiving (S) or not (NS) a nutritional supplementation of feed leftovers from a dairy cow enterprise (i.e., intensive system) in Northern Mexico.

This study was carried out in Northern Mexico (25° N, 103° W; 1148 m). The climate is BWhw″ (e′) according to the Köppen classification system [18], corresponding to very dry or desert climate that is semi-warm with rains in summer and cool in the winter, with extreme oscillations [19]. The mean annual temperature is 21.3 °C, reaching an extreme maximum temperature of 44 °C in May–June and an extreme minimum temperature of −2 °C during December–January [18]. The photoperiodic variations are 13:41 h of light during the summer solstice and 10:19 h during the winter solstice [20]. The average annual rainfall is 220 mm, with the highest rainfall occurring during summer and autumn [18]. The study area is in the central region of the Chihuahuan Desert, where the predominant vegetation includes shrubs, as well as grasses [21]. Goats were kept on rangeland from 1030 to 1900 h, with occasional grazing of crop residues (i.e., corn and sorghum); then, goats were housed overnight in pens, having access to mineral blocks, and drinking water *ad libitum* (Figure 1).

### 2.3. Animals and Their Management

*Male goats:* Crossbred dairy-type adult bucks (*n* = 4) of proven libido and fertility were used to induce estrus of goats managed under an extensive production system. Males were similar in BW and BCS (2.5; where 0 = emaciated, and 5 = obese). BCS was defined by palpation of the muscular and fat mass in the sternum and the transverse and spinous processes of the lumbar vertebrae, as formerly defined [22]. Bucks were previously separated from the female herd (i.e., 3 weeks) and induced to sexual activity by applying 50 mg of testosterone (Testosterone 50, Brovel^®^, Mexico City, Mexico) intramuscularly every third day for three weeks, from 6 to 27 March [23].

*Female goats*. Crossbred multiparous goats (*n* = 38) managed under rangeland conditions were diagnosed as anovulatory by ruling out the presence of corpora lutea (CL) by transrectal ultrasonography (Aloka SSD 500, Tokyo, Japan; 7.5 MHz transducer), on days −20, −10, and −1, considering day 0 (d0) the onset of the experimental breeding (28 March). During the deep-anestrous season (i.e., March–April, 25° N), multiparous goats with a similar body weight (BW) and body condition score (BCS), were randomized into two groups: (1) supplemented group (SG; *n* = 19; BCS: 1.76 ± 0.07; BW: 43.7 ± 1.8 kg), receiving 400 g goat d^−1^ of dairy cow feed leftovers (Table 1) prior to grazing; and (2) non-supplemented groups (NS; *n* = 19; BCS: 1.76 ± 0.06; BW: 44.3 ± 2.5 kg). Preceding the grazing activity, the short-term supplementation (i.e., 400 g) was individually offered to each goat in a plastic container with a capacity 5.0 kg. Previously, the feed leftovers from a dairy cow enterprise were collected daily at 07:00 and brought to the goat corrals to be offered to the SG goats. From d −5 to d +15 (i.e., 23 March to 12 April), both groups of goats grazed from 09:00 to 19:00 h. The sampling process of the feed leftovers was carried out by taking 10 samples (i.e., 50 g each) along the feeding line and subsequently analyzed the samples’ chemical and physical composition in the Chemistry Laboratory of the university (Table 1).

*Male effect*. On 28 March (d0) at 19:00 h, two bucks were introduced to each group of goats; bucks were exchanged between pens of goats every 12 h. Twenty-four hours before buck’s introduction, each goat received 25 mg of progesterone i.m. (Progesterone, Lab. Zoetis^®^, Mexico City, Mexico) to minimize the occurrence of short estrous cycles [26]. Additionally, the female’s BW was registered on days −5, −1, and +15, using a digital pendulum scale with a 150 kg capacity (Weiheng, Model: WH-C100, China). Blood glucose was also quantified on days −5, −1, and +15, considering a 12 h fasting period; a drop of blood was collected by venipuncture of the jugular and immediately analyzed in a portable glucometer (ABCSu-Chek ^®^ Roche, Model 0086, Basel, Switzerland) with a reliability level of 95%.

#### 2.3.1. Response Variables

*Estrus response.* The estrus response of goats was individually registered by exposing them to sexually active bucks from 19:00 to 09:00 h for 10 d. When they remained immobile and allowed the mount, goats were considered in estrus [27]. The latency to estrus was defined as the period (i.e., h) from the exposure to the bucks (d0) until the moment that goats accepted to be mounted. Regarding the variable duration of estrus, time was quantified from the first mount until the last one that each goat allowed during the experimental breeding.

*Reproductive response.* The proportion of goats that ovulated was quantified in each goat on d 10 of the experimental period (i.e., 8 April), throughout transrectal ultrasound examination (Aloka SSD-500, Tokyo, Japan; 7.5 MHz); no goat showed signs of estrus after day 10 of the experimental breeding. In both ovaries, the number of corpora lutea, as evidence of ovulation, was categorized as single, double, or triple. Every CL was measured to determine its luteal tissue volume (V) by using the following formula: V = 4/3 × π × r^3^. The radius (r) was calculated using the formula r = (L/2 + A/2)/2, where L = length, and A = width [28]. While the total number of ovulations was calculated in each group, the pregnancy rate was ultrasonographically diagnosed on day +36.

#### 2.3.2. Statistical Analysis

A completely randomized experimental design was used. Based on the results of the Kolmogorov–Smirnov test, parametric or non-parametric analyzes were performed for the different variables. The goats’ blood glucose values, BW, and BCS on days −5, −1, and +15 were compared with an ANOVA. Estrus latency was analyzed using a Kruskal–Wallis test; subsequently, the OR was compared using the Mann–Whitney U test; and, finally, the χ² test was used for data relating to proportions such as goats in estrus, ovulated goats, types of ovulation, and pregnant goats at day 36 after mating. All data were analyzed using SPSS 25 software (IBM SPSS Statistics for Windows, Version 25.0., Armonk, NY, USA: IBM Corp). The threshold to define a statistical significance was established at 0.05.

## 3. Results

The body weight and body condition score of both groups did not differ (*p* > 0.05) throughout the experimental period. Moreover, the blood glucose values followed the same trend between treatments across the study, with no differences across time (i.e., d −5, −1, and +15; *p* > 0.05) (Table 2). Regarding the estrus response (i.e., estrus induction, estrus latency, and estrus duration), no differences between experimental groups were detected in the study (*p* > 0.05) (Table 3); no short cycles were detected.

The proportion of goats ovulating (%), volume of luteal tissue (mm^3^), proportions of ovulation type (i.e., single, double, triple, or multiple), and pregnancy rate at d 45 after are shown in Table 3. With respect to the response variables, estrus induction, latency to estrus, estrus duration, and luteal tissue volume did not differ between experimental groups (*p* > 0.05). On the contrary, the phenotypic values for goats ovulating (78.94 vs. 47.36%), total number of corpora luteum (*n*), and ovulation rate differed (*p* < 0.05) between experimental groups, favoring to the SG goats. In addition, regarding the type of ovulation (i.e., simple, double, triple, or multiple), while no differences (*p* > 0.05) occurred in the percentage of single and triple ovulations between experimental groups, both the proportions of double (60.0 vs. 44.4%) and multiple (73.3 vs. 55.5%) ovulations favored (*p* < 0.05) to the SG goats. Moreover, the pregnancy rate diagnosed at d 36 post-mating (68.4 vs. 36.8%) also favored to the SG (Table 3).

## 4. Discussion

Based on a circular economy approach, our working hypothesis stated that a short-term supplementation with feed leftovers from dairy farms (i.e., intensive system) will increase the reproductive outcomes of rangeland managed goats (i.e., extensive system), trying to promote the circularity between two highly discordant ruminant production schemes. As proposed, the response variables number of goats ovulating, total number of corpuses luteum, ovulation rate, number of multiple ovulations, and pregnancy rate favored the supplemented goats. Thus, based on our main research outcomes, our working hypothesis was not rejected. As expected, the suggested circularity or connectedness between two extremely dissimilar ruminant production systems, throughout a kind of “*Robin Hood Effect*”, allowed the use of feed wastes of a highly industrialized large-scale dairy cow system to enhance the reproductive performance of a marginal–extensive goat production scheme that is more circular and less linear, resembling a more closed loop. That is, an attempt to re-signify animal production through new approaches of socioeconomic–ecological relationships with a clean–green–ethical emphasis (i.e., extract–produce–dispose vs. rethink–reduce–reuse). That was our main commitment.

The physiological processes of reproduction are energetically demanding and are regulated by energy balance, defined as the difference between feed availability and energy consumption, which influences the feedback mechanisms that regulate the hypothalamic–pituitary–gonadal reproductive axis [6]. In our study, the observed augments in the reproductive response of the SG were independent of either BW or BCS, along with the experimental period. Their independence from the experimental period could be expected, as the supplementation period lasted only 20 days. However, such a strategic supplementation certainly impacted, in a positive fashion, diverse reproductive outcomes, from goats ovulating up to goats pregnant. The acute effect of short-term supplementation, which does not promote changes in BW and BCS, may promote a positive effect on follicular growth and ovulation rate, being more evident in animals with low BW and BCS [29], as it has been documented that food supplementation stimulates follicular growth [30], and some studies reported that a short-term nutritional supplementation in sheep augmented the ovulatory activity [31]. The same trend was also observed in females receiving a short-term legume supplementation and displaying augmented ovulatory activity [32].

In the arid lands of Northern Mexico, a high percentage of kidding occurs in summer, denoting a breeding period around early winter. However, this period is unfavorable because it is the time of the year with the most significant food shortages. A strategy to manipulate reproduction more efficiently is to perform hormonal treatments in males (50 mg of testosterone every third day for 3 weeks) [23] so that mating begins in the first week of May. With this strategy, goats become pregnant in better conditions of food availability, and kidding occurs at the beginning of October, when the price of the kid is more favorable, and the availability of milk for sale is continuous throughout the year. In this arid region, environmental temperatures increase considerably; however, goats tend to be less sensitive to heat stress, and, therefore, the magnitude of the biological mechanisms of thermoregulation is reduced. As previously proposed, the reduction in the magnitude of the biological mechanisms of thermoregulation can be related to an augmented efficiency in the use of water and in the fiber digestion by goats [33], as is also seen in Kacang goats, a local breed well adapted to the extreme environment of the hot-dry region of Indonesia with a similar production scheme—grazing during the day and pen-sheltered at night [34].

Ovarian function can be influenced by high-in-energy and/or high-in-protein diets if such diets are provided for a few days just before ovulation [6,31,32]. In addition, ovulation induction by the stimulus of the male in anovulatory goats is modulated not only by the depth of the anestrus [35] but also the BW of goats [36]. However, in the present study, no difference was detected between groups when considering the estrus responses (i.e., estrus induction, latency to estrus, and estrus duration). This can be explained by the fact that goats, in times of scarce forage availability, show a particular reproductive strategy that is different from other farm animals, since most goats “respond” to the male effect regardless of the degree of body energy reserves [37]. Certainly, goats with low BCS “respond” to the male effect; however, this response is delayed and minor as compared to good body-conditioned goats [7]. Unlike with sheep, food restriction is not a significant limitation for goats to reestablish their reproductive activity [38]. Certainly, even goats at middle or low BCS can be induced to present estrus, to ovulate [39], to be able to conceive [40], and to carry their gestation until term [41]. It has been proposed that the insulin-mediated glucose uptake in ovarian cells is important for the reproduction of small ruminants since the gonadotropin-dependent follicular development can be modulated by numerous external influences, with insulin being one of them; in turn, insulin modulates the action of gonadotropins upon follicular growth [42]. Thus, maximizing the production of glucose precursors is fundamental to enhance reproductive success [43]. In the present study, no differences in blood glucose values occurred between groups across the experimental period (i.e., days −5, −1, and +15), and this result is coincident with increases in the ovulation rate without the modifications in blood glucose concentrations previously described [30].

The nutritional status of goats is intrinsically related to reproductive success; diverse metabolites and metabolic hormones have been indicated to act as enhancers of reproductive processes such as follicular and oocyte development, fertilization of ovum, and embryonic and fetal survivals, among others [44]. Interestingly, in our study, no differences in BW and BCS occurred between experimental groups or with the experimental period, yet a significant difference in the pregnancy rate at d36 after mating favored to the SG goats. Therefore, the short-term nutritional supplementation offered to the SG goats may activated some nutritionally dependent pathways, affecting, in a positive and direct fashion, ovarian function and enhancing the oviduct and uterine environments, which, in turn, allowed an increased pregnancy rate at d36 post-mating irrespective of BW or BCS. Among the most suitable metabolic hormone players modulating such an interesting response in the SG are growth hormone, insulin-like growth factor I, leptin, glucose, and insulin [44]. Furthermore, it has been reported that a nutritional supplementation before the fertilization period, either of short or medium duration, has a positive effect upon the out-of-season reproductive outcomes in goats exposed to the male effect [36,45].

Our study validates the feasibility of transferring a small fuel efflux from a dairy cow production system (i.e., intensive) towards the counter-season reproductive outcomes of a goat production system (i.e., extensive). Nonetheless, this supplementation strategy must be evaluated in regard to other productive outputs, such as health status, growth rates, milk yield, and fiber production in small ruminants. Interestingly, the region where our study was performed (i.e., Comarca Lagunera) has one of the largest industrialized/intensified dairy cattle hubs in The Americas, with an inventory close to 500,000 Holstein cows [14], which display a daily average intake of 26.5 kg per cow of a total mixed ration, representing a total daily intake of 13,068,316 kg. If we consider an average diet rejection of 4%, the potential feed leftovers of the intensive dairy cow production system signify more than 522,000 kg per day, which can be transferred to the less privileged extensive goat production system. That is, if projecting to an annual basis the theoretically available 522 tons of feed leftovers, the marginal–extensive goats production system would potentially receive a fuel influx of 190,530 tons yearly. Moreover, we need to notice that our study was performed under marginal conditions not only from a biotic and social but an economic standpoint, a recurrent scenario observed in most of the world areas where goat production occurs. Undoubtedly, there are exciting research opportunities ahead to enhance not only the livelihoods of goat keepers and their families but to better understand the trade-offs and ecological footprints between dissimilar ruminant production systems under fragile environments.

## 5. Conclusions

Considering the circular economy approach, we intended to evaluate the feasibility to offer a short-term supplementation with feed leftovers from dairy farms (i.e., intensive system) to increase the reproductive outcomes of rangeland managed goats (i.e., extensive system). The response variables number of goats ovulating, total number of corpuses luteum, ovulation rate, number of multiple ovulations, and pregnancy rate at d36 favored the supplemented goats compared to the control group. The proposed circularity or connectedness between two extremely dissimilar ruminant production systems, throughout a kind of “*Robin Hood Effect*”, allowed the use of feed wastes of a highly industrialized large-scale dairy cow system to enhance the reproductive performance of a marginal–extensive goat production scheme that is more circular and less linear, resembling a more closed loop. This research attempts to re-signify animal production through new approaches of socioeconomic–ecological relationships with a clean–green–ethical emphasis (i.e., extract–produce–dispose vs. rethink–reduce–reuse). At the end, this research effort intends to promote the enhancement of the animal ecological services, the animal well-being, and the livelihoods of the goat-producer and his family.

## Figures and Tables

**Table 1:** Chemical and physical composition of the dairy cow feed leftovers used as targeted short-term nutritional supplementation (20 days) to multiparous dairy crossbred goats managed under rangeland production scheme in arid Northern Mexico ^1^.

Ingredients ^2^ (g/kg DM^−1^)	
Corn silage	241.6
Rolled corn grain	310.7
Alfalfa hay	135.2
Soybean meal	123.5
Cottonseed meal	94.5
Vitamins and minerals premix	94.5
Nutrient content of diet (%/kg^−1^)	
Dry matter	80.0
Ash	7.3
Ether extract	3.8
Crude protein	15.3
Neutral detergent fiber	26.9
Acid detergent fiber	17.8
Total detergent nutrients	88.9
Metabolizable energy (Mcal/kg MS^−1^) ^3^	3.5

^1^ The short-term supplementation period included 20 days (i.e., d −5 to d +15 of the experimental breeding). ^2^ The presence of *Aspergillus flavus* in corn by-products was discarded [24]. ^3^ The metabolizable energy was calculated according to the equation proposed by the NRC (2001) [25].

**Table 2:** Means ± standard error for body weight (BW, kg), body condition score (BCS, units), and blood glucose level (mg/dL), on days −5, −1, and +15 of multiparous crossbred/rangeland goats (*n* = 38) (i.e., extensive system) receiving (S) or not (NS) a nutritional supplementation of feed leftovers from a dairy cow enterprise (i.e., intensive system) in Northern Mexico ^1^.

Variables ^2^	d − 5		d − 1		d + 15	
	SG	NSG	SG	NSG	SG	NSG
BW	43.7 ±1.85	44.3 ±2.50	43.8 ± 1.82	44.5 ±2.57	44.8 ± 1.80	45.3 ± 2.56
BCS	1.76 ± 0.07	1.76 ± 0.06	1.76 ± 0.07	1.76 ± 0.06	1.79± 0.07	1.76 ± 0.06
Glucose	60.2 ± 0.97	61.4 ± 1.58	60.0 ± 1.12	58.8 ± 1.07	64.0 ± 1.44	60.9 ± 1.46

^1^ The SG was supplemented from day −5 to +15 in relation to the mating period; d0 = onset of the experimental out-of-season breeding (i.e., 28 March to 7 April). SG = supplemented; NSG = non-supplemented. ^2^ For all the response variables, no differences were detected (*p* > 0.05).

**Table 3:** Frequencies and means ± standard error for estrus responses, ovulations (%), total number of corpuses luteum (*n*), ovulation rate (units), luteal tissue volume (mm^3^), type of ovulation (%), and pregnancy rate (d36, %) from multiparous crossbred/rangeland goats (*n* = 38) (i.e., extensive system) receiving (SG, *n* = 19) or not (NSG, *n* = 19) a nutritional supplementation of feed leftovers from a dairy cow enterprise (i.e., intensive system) in Northern Mexico ^1^.

Variables	Groups	
	SG	NSG
Estrus induction (%)	16/19 (84.21)	13/19 (68.42)
Latency to estrus (h)	99.75 ± 8.7	101.54 ± 12.3
Estrus duration (h)	32.25 ± 3.2	31.38 ± 4.0
Goats ovulating, *n* (%)	15/19 (78.94) ^a^	9/19 (47.36) ^b^
Total number of corpora luteum, (*n*)	27 ^a^	14 ^b^
Ovulation rate (*n*)	1.42 ± 0.23 ^a^	0.73 ± 0.21 ^b^
Luteal tissue volume (mm^3^)	657 ± 100	424 ± 89
Type of ovulation:		
-Single, *n* (%)	4/15 (26.67)	4/9 (44.44)
-Double, *n* (%)	9/15 (60.00) ^a^	4/9 (44.44) ^b^
-Triple, *n* (%)	2/15 (13.33)	1/9 (11.11)
-Multiple, *n* (%)	11/15 (73.33) ^a^	5/9 (55.55) ^b^
Pregnancy rate, d36, *n* (%)	13/19 (68.42) ^a^	7/19 (36.84) ^b^

^1^ The SG was supplemented from day −5 to +15 in relation to the mating period; d0 = onset of the experimental out-of-season breeding (i.e., 28 March to 7 April). SG = supplemented group; NS = non-supplemented group. ^a,b^ Response variables with different superscript between columns within row differ (*p* ≤ 0.05).

## Data Availability

None of the data were deposited in an official repository, yet information can be made available upon request.
